# Electrical brain imaging evidences left auditory cortex involvement in speech and non-speech discrimination based on temporal features

**DOI:** 10.1186/1744-9081-3-63

**Published:** 2007-12-10

**Authors:** Tino Zaehle, Lutz Jancke, Martin Meyer

**Affiliations:** 1 Department of Neuropsychology, University of Zurich, 8050 Zurich, Switzerland; 2 Institute of Neuroradiology, University Hospital of Zurich, 8091 Zurich, Switzerland

## Abstract

**Background:**

Speech perception is based on a variety of spectral and temporal acoustic features available in the acoustic signal. Voice-onset time (VOT) is considered an important cue that is cardinal for phonetic perception.

**Methods:**

In the present study, we recorded and compared scalp auditory evoked potentials (AEP) in response to consonant-vowel-syllables (CV) with varying voice-onset-times (VOT) and non-speech analogues with varying noise-onset-time (NOT). In particular, we aimed to investigate the spatio-temporal pattern of acoustic feature processing underlying elemental speech perception and relate this temporal processing mechanism to specific activations of the auditory cortex.

**Results:**

Results show that the characteristic AEP waveform in response to consonant-vowel-syllables is on a par with those of non-speech sounds with analogue temporal characteristics. The amplitude of the N1a and N1b component of the auditory evoked potentials significantly correlated with the duration of the VOT in CV and likewise, with the duration of the NOT in non-speech sounds.

Furthermore, current density maps indicate overlapping supratemporal networks involved in the perception of both speech and non-speech sounds with a bilateral activation pattern during the N1a time window and leftward asymmetry during the N1b time window. Elaborate regional statistical analysis of the activation over the middle and posterior portion of the supratemporal plane (STP) revealed strong left lateralized responses over the middle STP for both the N1a and N1b component, and a functional leftward asymmetry over the posterior STP for the N1b component.

**Conclusion:**

The present data demonstrate overlapping spatio-temporal brain responses during the perception of temporal acoustic cues in both speech and non-speech sounds. Source estimation evidences a preponderant role of the left middle and posterior auditory cortex in speech and non-speech discrimination based on temporal features. Therefore, in congruency with recent fMRI studies, we suggest that similar mechanisms underlie the perception of linguistically different but acoustically equivalent auditory events on the level of basic auditory analysis.

## Background

Auditory language perception is based on a variety of spectral and temporal acoustic information available in the speech signal [1]. One important temporal cue used to distinguish between stop-consonants is the voice onset time (VOT). The VOT, defined as the duration of the delay between release of closure and start of voicing, characterizes voicing differences among stop consonants in a wide variety of languages [2] and can thus be considered one of the most important acoustic cues encoding linguistically relevant information. The perceptual ability of resolving two signals as temporally discrete requires that the brain has a temporally segregated representation of those events.

Electrophysiological studies have consistently demonstrated VOT-related auditory evoked potential (AEP) differences in the N1 component with a single peak in response to short VOTs, and with a double-peaked in response to longer VOTs in humans [3-7], monkey [8,9] and guinea pig [10]. In humans it has been shown that non-speech sounds with related temporal characteristics as consonant-vowel-syllables (CV) resemble these pattern of acoustic temporal processing [11]. In particular, this study showed using intracerebral depth electrodes that the evoked responses of the left, but not the right primary auditory cortex are differential for the processing of voiced and voiceless consonants and their non-speech analogues.

Further support for a general mechanism for encoding and analysing successive temporal changes in acoustic signals has been evidenced by studies demonstrating that patients with acquired brain lesions and aphasia [12,13], children with general language-learning disabilities [14,15] and children and adults with dyslexia [16] show impaired auditory processing of temporal information in non-verbal stimuli. Furthermore, children with reading disabilities are deficient in phoneme perception, which is reflected by inconsistent labelling of tokens in VOT series [17,18], and these children also perform less consistently in labelling of tone onset time tokens [19] and exhibit poorer auditory order thresholds [20]. Moreover, it is known that the ability for phoneme discrimination in these children can be increased by a behavioural training using more salient versions of the rapidly changing elements in the acoustic waveform of speech [21,22].

Recent electrophysiological and neuroimaging studies point to the important role of the primary and secondary auditory cortex for the processing of acoustic features in speech and non-speech sounds. Several investigations using intracranial recording [9,11], scalp EEG [23,24], MEG [25] as well as fMRI [26,27] demonstrated an elevated role of the human primary auditory cortex for the temporal processing of short acoustic cues in speech and non-speech sounds. Furthermore, auditory association areas along the posterior supratemporal plane, in particular the bilateral planum temporale (PT) have also been associated with the processing of rapidly changing auditory information during sub-lexical processing [26,28,29]. However, due to BOLD-related limitations in temporal resolutions, the EEG method is far more suitable for elucidating the temporal organization of speech perception. In combination with a recently developed source estimation algorithm [30], it even allows the mapping the spatiotemporal dynamics of elemental aspects of speech perception, i.e. VOT decoding. Thus, the most important goal of this study is the validation of the aforementioned left middle and posterior auditory cortex recruitment in speech and non-speech discrimination based on temporal features.

In the present study, we recorded and compared scalp AEPs in response to CV-syllables and non-speech analogues with varying *VOT *and noise-onset-time (*NOT*), respectively. Here we aimed to investigate the neural coding of acoustic characteristics underlying speech perception and relate this temporal processing mechanism to specific activations of the auditory cortex. It has been demonstrated that these processing mechanisms are reflected by modulations of the AEP. The N1 deflection in particular is an obligatory component considered to reflect the basic encoding of acoustic information of the auditory cortex [31,32]. Furthermore, this component reflects the central auditory representation of speech sounds [33,34] and non-speech sounds [35]. Thus, in the context of the present study we focused on the modulations during the N1 time window elicited by brief auditory stimuli that varied systematically along an acoustic and a linguistic dimension. In addition, we examined the extent to which the pattern of neural activation differs in distinct portions of the auditory cortex. As mentioned above, both the middle compartment of the supratemporal plane (STP) accommodating the primary auditory cortex and the posterior compartment of the supratemporal plane harbouring the planum temporale are crucial for processing transient acoustic features in speech and non-speech sounds. In order to systematically investigate the contribution of these auditory cortex sections, we applied a low-resolution brain electromagnetic tomography (LORETA) approach and predicted functional leftward asymmetric responses to rapidly changing acoustic cues over the middle and posterior portion of the STP.

## Methods

**Figure 1:**
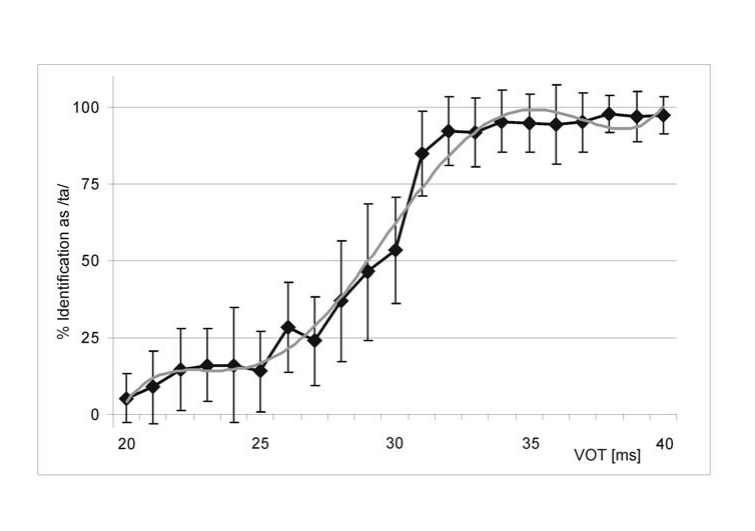
Averaged identification curve (+/-1 standard deviation) indicating the percentage of CV-syllables that were identified as /ta/ in relation to their VOT (black, diamonds) and fitted polynomial function (gray) [y = 0.0011x^5 ^- 0.059x^4 ^+ 1.0989x^3 ^- 8.0781x^2 ^+ 25.458x - 14.507]; Inflection point: x|y [10.98|63.86]; corresponding to a VOT of 29.98 ms.

In a behavioural pilot study, 24 healthy, right-handed native speakers of German (mean age = 26.7 ± 4.56 years, 13 female) performed a phonetic categorization task. A synthetic VOT continuum was used ranging from 20 to 40 ms VOT in 1 ms steps. Participants were instructed to listen to each syllable and to decide whether the syllable was [da] or [ta] by pressing a corresponding button as quickly and accurately as possible. Figure 1 illustrates results of this pilot study. The graph shows the averaged identification curve indicating the percentage of syllables that were identified as /ta/. As illustrated in Figure 1, the mean categorization boundary as indicated by the inflection point of the fitted polynomial function was at a VOT of 30 ms. The results of this behavioural study formed the basis for the subsequent electrophysiological investigation. As a consequence, we used syllables with a VOT of 5 ms, as they were consistently identified as the syllable /da/, a VOT of 60 ms, consistently identified as the syllable /ta/ and syllables with the VOT of 30 ms reflecting the averaged categorization boundary between /da/ and /ta/. We used a VOT of 5 ms for the voiced CV-/da/ and a VOT of 40 ms for the unvoiced CV-/ta/ to ensure the use of VOT stimuli that are clearly in the voiced segment (5 ms) and in the unvoiced segment (60 ms).

The electrophysiological experiment was conducted in a dimly lit, sound attenuated chamber. Subjects were placed in a comfortable chair at 110 cm distance from the monitor and scalp recorded event-related potentials (ERPs) in response to CV-syllables and non-speech sounds were obtained from 18 male right-handed, native German speaking healthy volunteers (mean age = 28.6 ± 3.45 years). None had any history of hearing, neurological, or psychiatric disorders. After a full explanation of the nature and risks of the study, subjects gave their informed consent for the participation according to a protocol approved by the local ethics committee.

**Figure 2:**
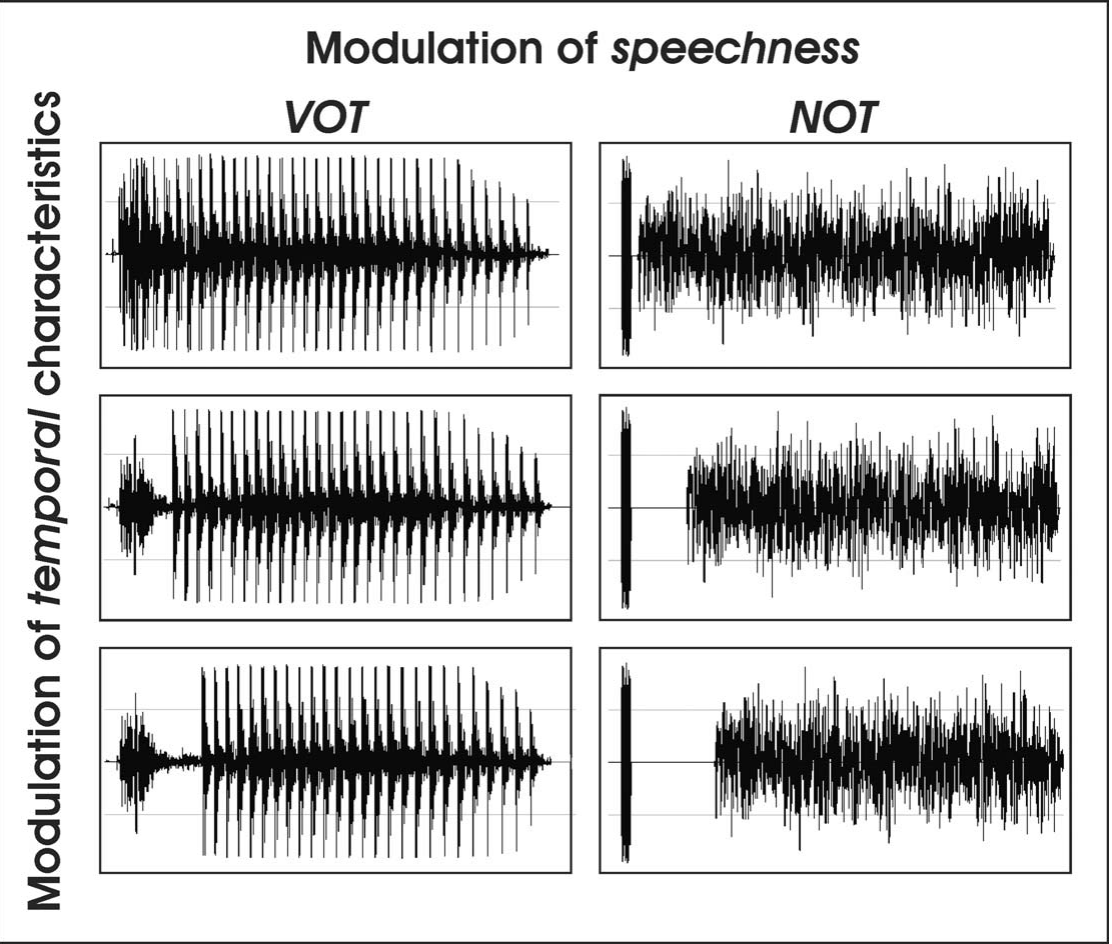
Waveforms of the auditory stimulation. The left panel shows speech stimuli (CV) with varying VOT (5, 30, 60 ms), and the right panel shows non-speech stimuli with varying NOT (top to bottom: 5, 30, 60 ms).

The auditory stimuli were generated with a sampling depth of 16 bits and a sampling rate of 44.1 kHz using the SoundForge 4.5 Software [36] and PRAAT [37]. We used a modified version of the stimulus material described by Zaehle et al., (2004) [26]. Figure 2 shows wave-forms of the applied stimuli. Stimuli material consisted of CV syllables with varying voice-onset-times (5 ms, 30 ms and 60 ms) as revealed in the pilot behavioural study and analogously, non-speech sounds with varying noise-onset-times (5 ms, 30 ms and 60 ms). For the non-speech condition, we created stimuli containing two sound elements separated by a gap. The leading element was a wideband noise burst with a length of 7 ms. The trailing element was a bandpassed noise centred on 1.0 kHz and a width of 500 Hz. The duration of the gap was varied. The duration of each single stimulus was consistent (330 ms). Auditory stimuli were presented binaurally using hi-fi headphones (55 dB sound pressure level). Stimulation and recording of the responses were controlled by the Presentation software (Neurobehavioral Systems, USA).

The EEG experiment comprised ten blocks. Within each block, 18 trials of each stimulus category were presented in a randomized order resulting in presentations of 180 stimuli-pairs. For each trial, volunteers performed a same-different discrimination task on a pair of stimuli belonging to one stimulus category. The stimuli varied with respect to the temporal manipulation of the *NOT *and *VOT*. Stimuli of one pair were presented with an inter stimulus interval of 1300 ms. Participants indicated their answers by pressing one of two response buttons. We utilized this task to ensure subjects' vigilance throughout the experiment and to engage the subjects to attend to the auditory stimulation. However, we were primarily interested in the electrophysiological responses to acoustic features underlying pure and elemental speech perception. We also aimed to avoid confounds with the neural correlates of decision making instantly following the second stimulus of each pair of VOT and NOT. Thus, only the first stimulus of each stimulus pair was analysed and included into the following analysis.

EEG was recorded from 32 scalp electrodes (30 channels + 2 eye channels) located at standard left and right hemisphere positions over frontal, central, parietal, occipital, and temporal areas (subset of international 10/10 system sites: Fz, FCz, Cz, CPz, Pz, Oz, Fp1, Fp2, F3, F4, C3, C4, P3, P4, O1, O2, F7, F8, T7, T8, P7, P8, TP7, TP8, FT7, FT8, FC3, FC4, CP3, and CP4) using a bandpass of 0.53 -70 Hz with a sampling rate of 500 Hz. We applied sintered silver/silver chloride electrodes (Ag/AgCl) and used the FCz position as the reference. Impedances of these electrodes were kept below 5 kΩ. Trials containing ocular artefacts, movement artefacts, or amplifier saturation were excluded from the averaged ERP waveforms. The processed data were re-referenced to a virtual reference derived from the average of all electrodes. Each ERP waveform was an average of more than 100 repetitions of the potentials evoked by the same stimulus type. The EEG recordings were sectioned into 600 ms epochs (100 ms pre-stimulus and 500 ms post-stimulus) and a baseline correction using the pre-stimulus portion of the signal was carried out. ERPs for each stimulus were averaged for each subject and grand-averaged across subjects.

In order to statistically confirm the predicted differences between AEP components at Cz as a function of experimental stimuli, mean amplitude ERPs time-locked to the auditory stimulation were measured in two latency windows (110–129 ms and 190–209 ms) determined by visual inspection covering the prominent N1a and N1b components. Analyses of variance (ANOVAs) with factors *temporal modulation *(5, 30, 60 ms) and *speechness *(VOT/NOT) were computed for central electrode (Cz), and the p values reported were adjusted with the Greenhouse-Geisser epsilon correction for nonsphericity.

Subsequently, we applied an inverse linear solution approach – LORETA (low-resolution electromagnetic tomography) to estimate the neural sources of event-related scalp potentials [38,39]. In order to verify the estimated localization of the N1a and N1b component, we calculated the LORETA current density value (μA/mm^2^) for the AEPs within the 3D voxel space. We used a transformation matrix with high regularization (1e3 * (first eigenvalue)) to increase signal to noise ratio. The maxima of the current density distributions were displayed on a cortical surface model and transformed in stereotactic Talairach space [40]. Subsequently, to specifically test the neurofunctional hypothesis of the bilateral middle and posterior STP, we calculated a post hoc region-of-interest (ROI) analysis. We defined four 3D ROIs in STP (left middle STP, right middle STP, left posterior STP, right posterior STP). The landmarks of ROIs were determined by an automatic anatomical labelling procedure implemented in LORETA. We collected mean current density values from each individual and each distinct 3D ROI by means of the ROI extractor software tool [41]. The mean current density values for each ROI were submitted to a 3 × 2 × 2 ANOVA with the factors *temporal modulation *(5, 30, 60 ms), *hemisphere *(left/right) and *speechness *(VOT/NOT)

## Results

**Figure 3:**
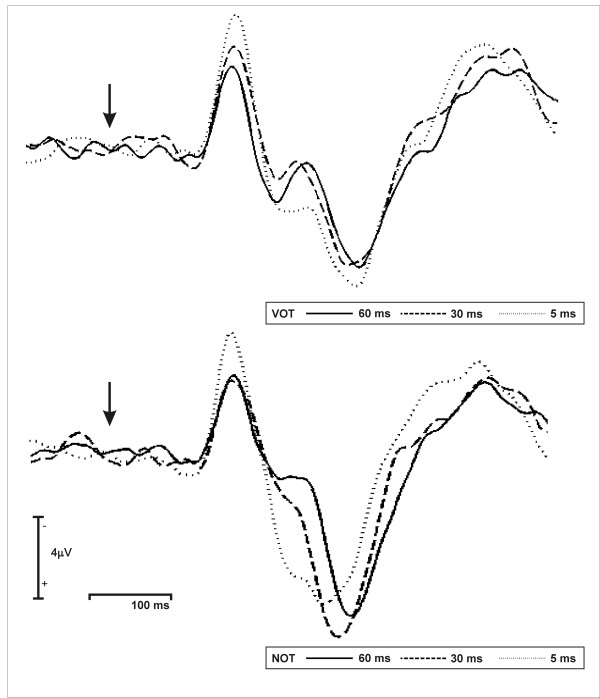
Averaged electrophysiological data, recorded from 18 participants time locked at the onset of stimulation at central (Cz) electrode during the perception of VOT (top) and NOT stimuli.

Grand averaged waveforms evoked by each of the three speech and three non-speech stimuli recorded from Cz are shown in Figure 3. We observed that all stimuli elicited a prominent N1a component with the shortest VOT/NOT modulation (5 ms) yielding the most enhanced amplitude. Furthermore, we noticed a second negative deflection peaking around 200 ms after stimulus onset (N1b) also revealing sensitivity to the temporal modulation of the sounds. In order to statistically examine the ERP effects, mean amplitude of the ERP waveforms were measured in two 20 ms latency windows.

Results of the 3 × 2 ANOVA with the factors *temporal modulation *(5, 30, 60 ms) and *speechness *(VOT/NOT) for the N1a (TW I: 110–129 ms latency window) revealed a significant main effect of the factor *temporal modulation *(F(1.77, 30.1) = 12.45, p < 0.001). Similarly, the N1b (190–209 ms latency window) ANOVA revealed a significant main effect of the factor *temporal modulation *(F(1.58, 26.92) = 15.7, p < 0.001). Furthermore, the ANOVA for the N1b also revealed a significant main effect of the factor *speechness *(F(1, 17) = 19.88, p < 0.001) and a significant *temporal modulation *by *speechness *interaction (F(1.6, 27.4) = 4.79, p < 0.05).

**Figure 4:**
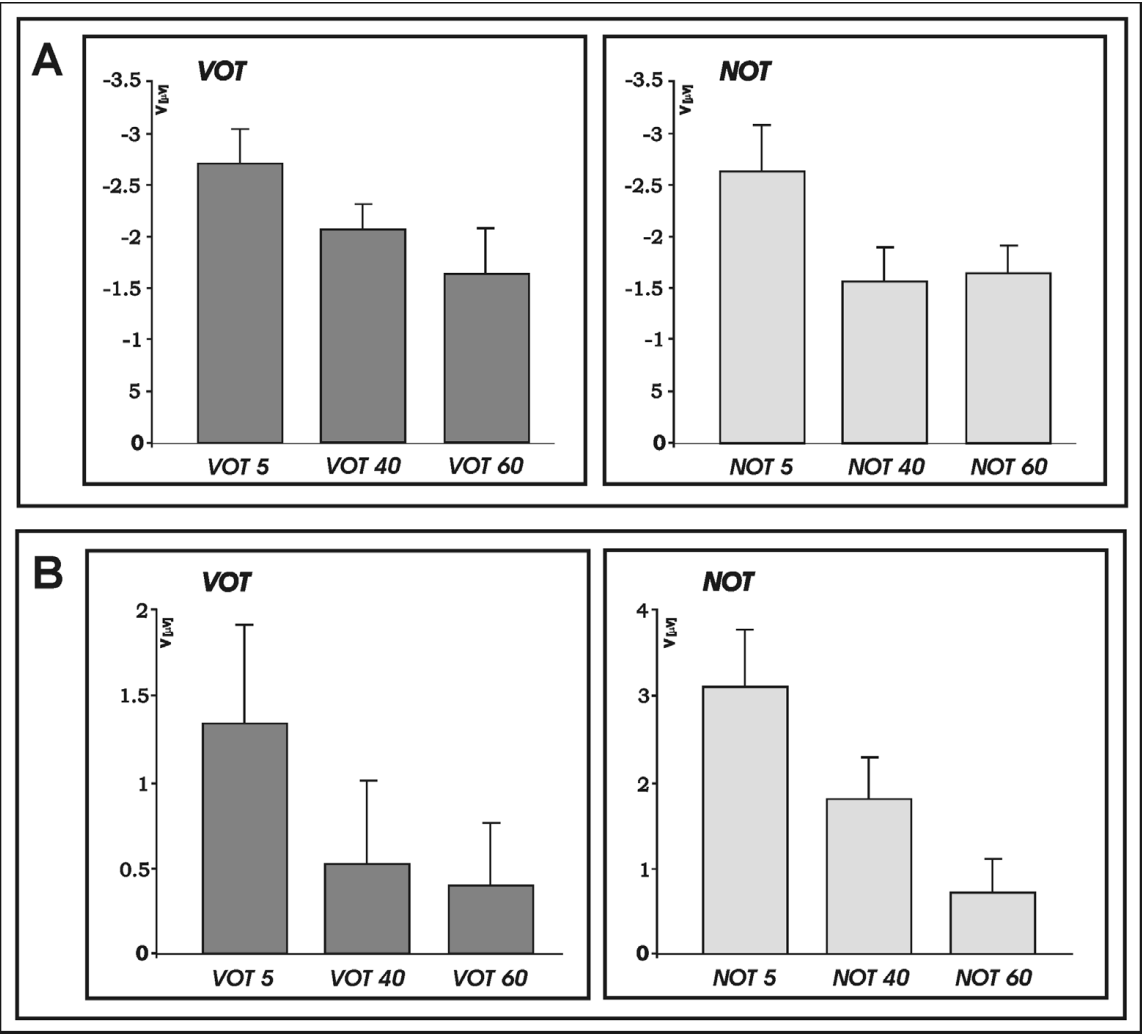
**a: **Plots of mean amplitude for N1a separate for *VOT *and *NOT *stimuli. **b: **Plots of mean amplitude for N1b separate for *VOT *and *NOT *stimuli.

Subsequently, *post-hoc *analyses were conducted separately for the speech and non-speech stimulation. Figure 4 shows plots of mean amplitude of the temporal modulation separated for speech and non-speech for a) N1a and b) N1b. The results of the one-factorial ANOVAs are listed in Table 1. For the N1 (110–129 ms latency), separate one-factorial ANOVA revealed a significant main effect of the factor *temporal modulation *for the non-speech sounds (F(1.8, 30.9) = 8.14 p < 0.001). Test for linear contrast demonstrated a significant linear relationship of the N1a mean amplitude and length of the *NOT *in the non-speech sounds (F(1,17) = 15.53, p = 0.001). Similarly, one – factorial ANOVAs with the factor *temporal modulation *in the speech sounds revealed a significant main effect (F(1.61, 27.4) = 5.34, p < 0.05) and test for linear contrast revealed significant linear relationship of the N1a mean amplitude and length of the *VOT *in the speech sounds (F(1,17) = 9.39, p < 0.05). The same pattern of activation was present at the 190 – 209 ms latency window (N1b). Separate one-factorial ANOVAs revealed a significant main effect of the factor *temporal modulation *for the non-speech sounds (F(1.23, 21.1) = 18.09, p < 0.001), and a one-factorial ANOVA with the factor *temporal modulation *revealed a significant main effect (F(1.79, 30.49) = 3.85, p < 0.05) for the speech sounds. Tests for linear contrast revealed a significant linear relationship of the N1b mean amplitude and length of the *NOT *in the non-speech sounds (F(1,17) = 24.18, p < 0.001), and *VOT *in the speech sounds (F(1,17) = 4.99, p < 0.05).

**Table 1:** Results of ANOVAs with the factor NOT and VOT for TW I and TW II

**Factor**				**linear contrast**		
	**df**	**F-value**	**p-value**	**df**	**F-value**	**p-value**

Time window I (N1)						
*VOT*	1.61	5.34	0.01	1	9.39	0.007
*NOT*	1.81	8.14	0.001	1	15.53	0.001

Time window II (N2)						
*VOT*	1.79	3.84	0.03	1	4.98	0.04
*NOT*	1.24	18.09	0.000	1	24.18	0.000

**Figure 5:**
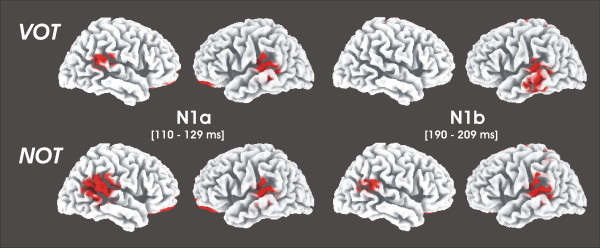
Grand average (n = 18) three dimensional LORETTA – based current density maxima for AEP components N1 and N2. (Threshold: 0.001 prop. μA/mm^2^).

Results for the source localization analysis are presented in Table 2. The table lists coordinates and corresponding brain regions associated with current density maxima for the speech and non-speech sounds obtained separately for the N1a and N1b time windows. As shown in Figure 5, for the N1a time window current density maps indicate that left and right posterior perisylvian areas contribute to both speech and non-speech sounds. With regard to the N1b, source estimation showed enlarged current density distribution over the left posterior STP and the anterior cingulate gyrus for speech and non-speech sounds, and the right posterior STP for non-speech sounds.

**Table 2:** Current density maxima [μA/mm^2^]*10^-3 ^in response to speech (VOT) and non-speech (NOT) sounds

Component	Condition	Brain Region	Current density value	Hemisphere	X	Y	Z
N1a	VOT	Cingulum	1.74		-3	45	1
		STG	1.39	L	-59	-32	8
			1.30	R	60	-39	15
							
	NOT	Cingulum	2.70		-3	45	1
		STG	1.50	L	-59	-32	8
			1.78	R	60	-39	15
							
N1b	VOT	Cingulum	1.74		-3	45	1
		STG	1.39	L	-59	-32	8
							
	NOT	Cingulum	2.70		-3	52	1
		STG	1.50	L	-59	-32	8
			1.78	R	60	-39	15

**Figure 6:**
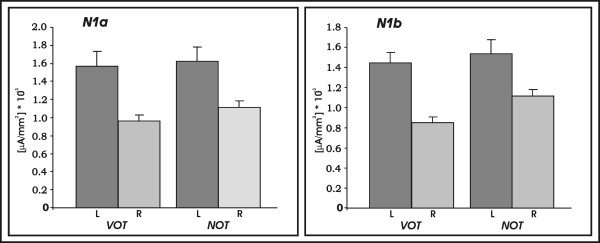
Plots of mean current density values obtained by the anatomically defined ROI analysis, separate for the left and right middle portion of the supratemporal plane (BA41): Left panel shows date for N1a (TW I) and the right panel shows data for N1b (TW II).

Subsequent statistical analysis of ROIs over the bilateral middle portion of the STP separate for N1a and N1b time windows revealed that current density values were strongly lateralized. A 3 × 2 × 2 ANOVA with the factors *temporal modulation *(5, 30, 60 ms)*, hemisphere *(left/right) and *speechness *(VOT/NOT) revealed a significant main effect of the factor *hemisphere *(F(1,17) = 18.64, p < 0.001) for the N1a as well as for the N1b time window (F(1,17) = 27.97, p < 0.001) demonstrating stronger responses over the left as compared to the right primary auditory cortex. Figure 6 shows current density values during the processing of VOT and NOT stimuli collapsed over the temporal modulations and extracted from the left and right primary auditory cortex.

**Figure 7:**
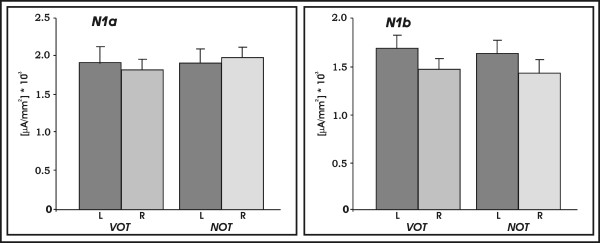
Plots of mean current density values obtained by the anatomically defined ROI analysis, separate for the left and right posterior portion of the supratemporal plane (post BA42): Left panel shows date for N1a (TW I) and the right panel shows data for N1b (TW II).

The analysis for the posterior portion of the STP showed no significant main effect or an interaction for the N1a time window. For the N1b time window, analysis showed a significant main effect of the factor *hemisphere *(F(1,17) = 5.55, p < 0.05) indicating stronger responses over the left as compared to the right posterior STP. Figure 7 shows current density values during the processing of VOT and NOT stimuli extracted from the left and right posterior portion of the STP.

## Discussion

One of the key questions in understanding the nature of speech perception is to what extent the human brain has unique speech-specific mechanisms or to what degree it processes sounds equally depending on their acoustic properties. In the present study we showed that the characteristic AEP waveform in response to consonant-vowel-syllables shows an almost identical spatio-temporal pattern as in response to non-speech sounds with similar temporal characteristics. The amplitudes of the N1a and N1b component of the auditory evoked potentials significantly correlated with the duration of the VOT in CV-syllables and analogously, with the duration of the NOT in non-speech sounds. Furthermore, current density maps of the N1a and N1b time windows indicate overlapping neural distribution of these components originating from the same sections over the superior temporal plane that accommodates auditory cortex. For the analysis of the middle portion of the STP incorporating the primary auditory cortex, we revealed asymmetric activations that point to a stronger involvement of left supratemporal plane regardless of TW, speechness or temporal modulation. For the posterior part of the STP, the analysis of the current density values revealed a bilateral activation pattern during the N1a time window and a leftward asymmetry during the N1b time window for both the perception of speech and non-speech sounds.

In general, our data are in line with former electrophysiological studies investigating the processing of brief auditory cues but delivers novel insight in that it demonstrates a strong preference of the left middle and posterior auditory cortex for rapidly modulating temporal information by means of a low-resolution source estimation approach. Using MEG, it has been demonstrated that the AEP response to speech sounds exhibits an N100m, which is followed by a N200m at around 200–210 ms [42]. It has been proposed that the N200m is specific to acoustic parameters available in vowels, since acoustic, rather than phonetic, features of the stimulus triggered the N200m. Sharma and colleagues showed that the typical change in the AEP waveform morphology from single to double peaked N1 components is not a reliable indicator of perception of voicing contrasts in syllable-initial position [3]. In other words, a double-peak onset response cannot be considered a cortical correlate of the perception of voicelessness. Rather, it depends on the acoustic properties of the sound signal. For the perception of consonants with the same place of articulation, the critical acoustic feature that distinguishes between these consonants is the time between the burst at consonant initiation and the onset of voicing (VOT). Similarly, in the case of non-speech sounds the critical acoustic feature is the time (silent gap) between the trailing and leading noise elements. In both cases the ability to perform the task requires the listener to perceptually segregate the two sounds (or their onsets) in time, which in turn requires that the brain have temporally segregated responses to the two events (or their onsets) [43]. As demonstrated by the present data, overlapping cortical excitement was found for the detection of temporal cues in both speech and non-speech sounds. Therefore, our data support the notion of similar mechanisms underling the perception of auditory events that are equal in temporal acoustic structure but differ in their linguistic meaning.

It has been suggested that the primary auditory cortex is specifically involved in the perceptual elaboration of sounds with durations or spacing within a specific temporal grain [43] and this suggestion has been confirmed by studies demonstrating that primary auditory cortex evoked responses reflect encoding of VOT [9,11,23,24]. Furthermore, Heschl's gyrus (HG) is known to display a leftward structural asymmetry [44-47]. This asymmetry is related to a larger white matter volume of the left as compared to the right HG [44,48], as well as to asymmetries at the cellular level [49-52]. It has been hypothesized that this leftward asymmetry of the HG is related to a more efficient processing of rapidly changing acoustic information, which is relevant in speech perception [53].

The posterior part of the left STP that partly covers the planum temporale (PT) has also been associated with competence to mediate spectro-temporal integration during auditory perception [54,55]. In particular, the left posterior auditory cortex plays a prominent role when speech relevant auditory information has to be processed [26,27,56]. Akin to the primary auditory cortex that resides in HG, the posterior STP also has structural leftward asymmetry [57,58], which indicates a relationship between this brain region and the leftward lateralized specific functions relevant to speech perception.

The present study revealed a clear asymmetrical response pattern over the posterior supratemporal plane during the N1b (TW II) for both the NOT and the VOT condition. Interestingly, we also observed a symmetrical response pattern during the N1a component (TW I) over the same cortical portion. In this vein are the findings of Rimol and colleagues who reported that the well established right-ear advantage (REA, indicative of a left hemisphere superiority) during a dichotic listening (DL) syllable task is found to be significantly affected by VOT [59]. More elaborately, the authors compellingly demonstrate that the REA reverses into a left-ear advantage under certain constellations of different VOT in the DL tasks. In addition, a recent study applying LORETA source estimation revealed differentially lateralized responses over the posterior STP contingent upon constellations of different VOT using the same DL task [24]. Thus, it can be concluded that the degree of asymmetry during DL is influenced by the length of the VOT as evidenced by both behavioural and electrophysiological measures. Based on these findings it could be assumed that the early symmetric effect over the posterior STP might be related to the differentially asymmetric effects of VOT length since our source estimation approach did not specifically emphasize this effect.

As mentioned above, a long lasting question in auditory speech research concerns the nature of the VOT cue and asks to what extent the VOT is processed by specialized speech mechanisms or by more basic acoustically tuned mechanisms [60]. Evidence for a specialized speech processing stems from the well known observation that the perception of series of (synthetic) speech stimuli varying continuously in VOT is almost categorical [61]. This effect of categorical perception implicates that for a series of stimuli the percept exists only in one of two categories: the voiced and voiceless stop. Furthermore, listeners can discriminate differences in VOT considerably better when two stimuli lie in different phonetic categories than when the two stimuli are from the same category. However, the effect of categorical perception also exists for non-speech stimuli [60]. As suggested by Phillips (1993), as far as the stimulus representation in the primary auditory cortex is concerned, speech may be "special" only in the sense that spoken language is the most obvious stimulus in which the identification of the elements is dependent on temporal resolution [43]. In fact, data of the present study evidence that the middle and posterior auditory cortex especially of the left hemisphere is significantly involved in the processing of the acoustical features critical for the processing of temporal cues in both speech and non-speech sounds.

This conclusion corroborates recent fMRI research, but in addition demonstrates that EEG in combination with low-resolution tomography could be considered an ideal alternative to map the spatio-temporal patterns of speech perception. In a way, this approach outperforms the fMRI technology because it evidently demonstrates the temporal subtlety of elemental acoustic processing reflected by differential sensitivity and neural distribution of succeeding N1a and N1b responses to brief speech and speech-like stimuli. Of course, one should bear in mind that spatial resolution of electrophysiologically based localization methods is inferior to modern brain imaging techniques. Thus, one should by no means feel tempted to interpret the activation maps provided by LORETA in an fMRI-like manner. However, it has been proven that low-resolution tomography is capable of reliably distinguishing between sources originating from distinct sections of the superior temporal region [62]. This holds particularly true if low-resolution tomography is used to examine electrophysiological responses emerging from the left or right hemispheres [63].

## Conclusion

In essence, the present study delivers further evidence for the prominent role of the middle and posterior left supratemporal plane in the perception of rapidly changing cues, which is thought to be an essential device underlying speech perception [53,64,65].

## Authors' contributions

TZ designed the experimental paradigm, performed the data acquisition and statistical analysis and drafted the manuscript

LJ contributed to the hypothesis, design, results, discussion, and to the preparation of the manuscript

MM conceived of the study, participated in its design and coordination and contributed to the manuscript

All authors read and approved the final manuscript.
